# Pharmacological Evidence That *Dictyostelium* Differentiation-Inducing Factor 1 Promotes Glucose Uptake Partly via an Increase in Intracellular cAMP Content in Mouse 3T3-L1 Cells

**DOI:** 10.3390/molecules28237926

**Published:** 2023-12-04

**Authors:** Yuzuru Kubohara, Yuko Fukunaga, Haruhisa Kikuchi, Hidekazu Kuwayama

**Affiliations:** 1 Laboratory of Health and Life Science, Graduate School of Health and Sports Science, Juntendo University, Inzai 270-1695, Japan; 2 Department of Animal Risk Management, Faculty of Risk and Crisis Management, Chiba Institute of Science, Choshi 288-0025, Japan; yfukunaga@cis.ac.jp; 3 Division of Natural Medicines, Faculty of Pharmacy, Keio University, Tokyo 105-8512, Japan; halkiku@keio.jp; 4 Graduate School of Life and Environmental Sciences, University of Tsukuba, Tsukuba 305-8572, Japan; kuwayama.hidekazu.fu@u.tsukuba.ac.jp

**Keywords:** *Dictyostelium discoideum*, DIF-1, obesity, diabetes, PDE1, cAMP, forskolin

## Abstract

Differentiation-inducing factor 1 (DIF-1) isolated from the cellular slime mold *Dictyostelium discoideum* can inhibit mammalian calmodulin-dependent cAMP/cGMP phosphodiesterase (PDE1) in vitro. DIF-1 also promotes glucose uptake, at least in part, via a mitochondria- and AMPK-dependent pathway in mouse 3T3-L1 fibroblast cells, but the mechanism underlying this effect has not been fully elucidated. In this study, we investigated the effects of DIF-1 on intracellular cAMP and cGMP levels, as well as the effects that DIF-1 and several compounds that increase cAMP and cGMP levels have on glucose uptake in confluent 3T3-L1 cells. DIF-1 at 20 μM (a concentration that promotes glucose uptake) increased the level of intracellular cAMP by about 20% but did not affect the level of intracellular cGMP. Neither the PDE1 inhibitor 8-methoxymethyl-3-isobutyl-1-methylxanthine at 10–200 μM nor the broad-range PDE inhibitor 3-isobutyl-1-methylxanthine at 40–400 μM had any marked effects on glucose uptake. The membrane-permeable cAMP analog 8-bromo-cAMP at 200–1000 μM significantly promoted glucose uptake (by 20–25%), whereas the membrane-permeable cGMP analog 8-bromo-cGMP at 3–100 μM did not affect glucose uptake. The adenylate cyclase activator forskolin at 1–10 μM promoted glucose uptake by 20–30%. Thus, DIF-1 may promote glucose uptake by 3T3-L1 cells, at least in part, via an increase in intracellular cAMP level.

## 1. Introduction

**Figure 1:**
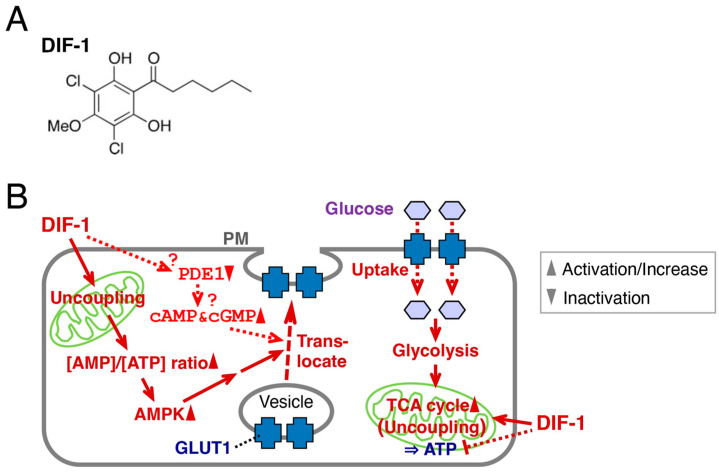
(**A**) Chemical structure of DIF-1. (**B**) Proposed scheme for the mechanisms of action of DIF-1. DIF-1–mediated uncoupling of mitochondrial activities may disrupt ATP production and activate AMP kinase, which may then induce GLUT1 translocation to the plasma membrane (PM) and glucose uptake [23,24]. Glucose may be metabolized immediately via glycolysis and via the TCA cycle [24,25]. DIF-1 may also inhibit PDE1 [26] and thereby increase intracellular cAMP and/or cGMP levels, which might promote glucose uptake [29,30].

The cellular slime mold *Dictyostelium discoideum* is an excellent model organism for the study of cell and developmental biology, including cell division, cell differentiation, cell death, chemotaxis, and morphogenesis, because of its simple lifecycle; it forms fruiting bodies, each consisting of spores and a multicellular stalk at the completion of its development [1,2,3]. Differentiation-inducing factor 1 (DIF-1) (Figure 1A), a chlorinated alkylphenone expressed in *D. discoideum* during development, induces stalk cell differentiation [4,5,6] and modulates chemotactic cell movement [7,8].

Studies conducted since the 1990s have shown that DIF-1 and its derivatives have antitumor (anti-proliferative and anti-metastatic) activities in mammalian cells in vitro and in vivo [9,10,11,12,13,14,15,16,17,18,19,20,21,22], and thus DIF derivatives are expected to be lead compounds for the development of anticancer agents [20]. DIF-1 can also promote glucose uptake (consumption) in mammalian cells, such as mouse 3T3-L1 fibroblasts and 3T3-L1 adipocytes [23]. DIF-1 promotes glucose uptake by inducing translocation of glucose transporter 1 (GLUT1) from intracellular vesicles to the plasma membrane, in part via a phosphatidylinositol 3-kinase (PI3K)/Akt-independent pathway [23], namely, a mitochondria- and 5′-AMP-activated kinase (AMPK)-dependent pathway [24] (Figure 1B). Oral administration of DIF-1 decreased blood glucose in streptozotocin-treated diabetic rats [25]. Thus, DIF-1 and its derivatives are potential platforms for the development of novel obesity and diabetes therapies. Despite details of how these molecules exert their actions remaining unclear (Figure 1B), it has been suggested that DIF-1 directly inhibits PDE1 [26], a calmodulin-dependent phosphodiesterase (PDE) that degrades cAMP and cGMP, which might increase intracellular cAMP and cGMP levels in various mammalian cells [27,28]. In K562 human leukemia cells, DIF-1 dose-dependently raised intracellular cAMP levels, whereas PDE inhibitors dose-dependently suppressed cell growth, suggesting that DIF-1 may suppress cell growth, at least in part, via PDE inhibition and a subsequent rise in intracellular cAMP [26].

Here, to further elucidate the mechanism(s) underlying the glucose-uptake-promoting effect of DIF-1, we analyzed the relationship between intracellular cAMP and cGMP levels and DIF-1 activity in confluent 3T3-L1 fibroblasts. We found that DIF-1 may promote glucose uptake partly via an increase in intracellular cAMP level in these cells.

## 2. Results

### 2.1. Effects of DIF-1 on Glucose Uptake and Cell Growth in 3T3-L1 Cells

**Figure 2:**
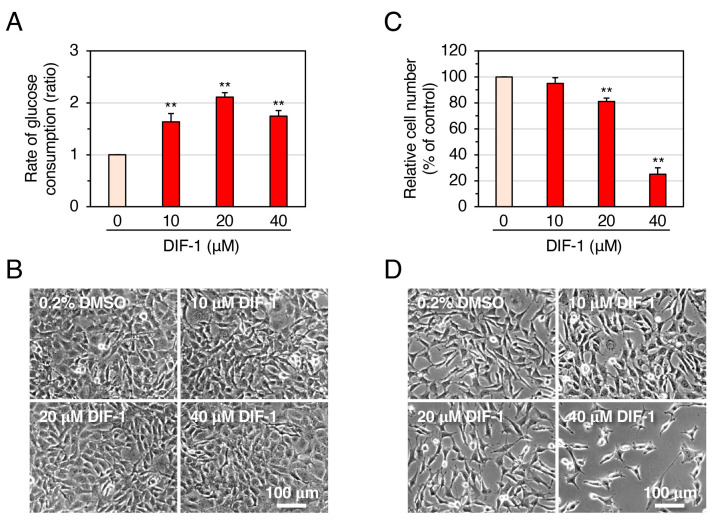
Effects of DIF-1 on glucose uptake (**A**,**B**) and growth of 3T3-L1 cells (**C**,**D**). (**A**) Confluent 3T3-L1 cells were incubated for 16–20 h in the presence of 0.2% dimethyl sulfoxide (DMSO; vehicle) or 10–40 μM DIF-1, and the rate of glucose consumption was assessed. The data are mean ± SD of three independent experiments. ** *p* < 0.01 versus control (by *t*-test). Note that the rate of glucose consumption measured by our method matches well with that of glucose uptake assessed with 2-[1,2-^3^H]deoxy-d-glucose [23]; therefore, sometimes we refer to ‘glucose consumption’ as ‘glucose uptake’. (**B**) Confluent 3T3-L1 cells were incubated as in (**A**) and observed under a phase-contrast microscope; representative photos are shown. (**C**) Growing 3T3-L1 cells were incubated for 3 days in the presence of 0.2% DMSO (vehicle) or 10–40 μM DIF-1, and relative cell number was determined. The data are mean ± SD of three independent experiments. ** *p* < 0.01 versus control (by *t*-test). (**D**) Growing 3T3-L1 cells were incubated for 3 days as in (**C**) and observed under a phase-contrast microscope; representative photos are shown.

We first examined the effects of DIF-1 (10–40 μM) on glucose uptake and cell growth in 3T3-L1 cells (Figure 2). DIF-1 at 10 or 20 μM dose-dependently increased the rate of glucose consumption, consistent with our previous report [23], in confluent 3T3-L1 cells (Figure 2A), whereas no dose dependence was observed with 40 μM DIF-1 (Figure 2A). As far as cell morphology is concerned, no cytotoxicity of DIF-1 was observed within the dose range examined (Figure 2B); note that most cells (>95%) remained viable after 24 h incubation with 100 μM DIF-1 [31]. DIF-1 at 10–40 μM dose-dependently suppressed cell growth in 3T3-L1 cells (Figure 2C,D), as expected from previous reports [18,32]. DIF-1 at 10 or 20 μM strongly promoted glucose uptake, but its anti-proliferative activity was weak, which supports our expectation that DIF-1 could be a good lead compound for the development of anti-diabetes and/or anti-obesity drugs rather than antitumor drugs.

### 2.2. Effects of DIF-1 on Intracellular cAMP and cGMP Levels in Confluent 3T3-L1 Cells

**Figure 3:**
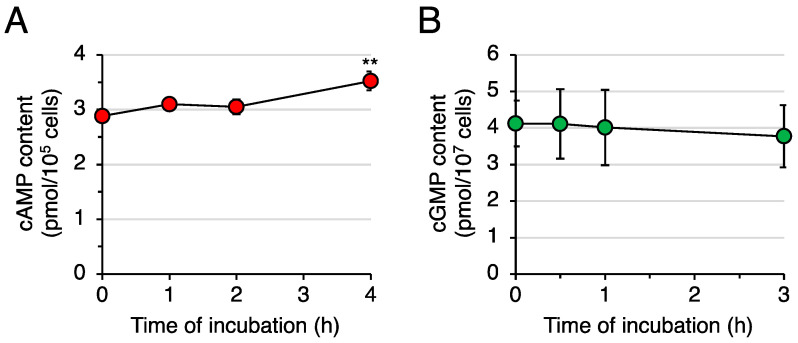
Effects of DIF-1 on intracellular cAMP and cGMP contents in confluent 3T3-L1 cells. Cells were incubated in the presence of 20 μM DIF-1, harvested at the indicated time points, and assayed for (**A**) cAMP or (**B**) cGMP content. The data are mean ± SD of triplicate samples in a single experiment. ** *p* < 0.01 versus Time 0 control (by *t*-test).

To test whether DIF-1 promotes glucose uptake via PDE1 inhibition, we examined the effects of DIF-1 on intracellular cAMP and cGMP levels in confluent 3T3-L1 cells (Figure 3). Since glucose uptake promoted by DIF-1 reaches its maximum at 3–4 h after the addition of 20 μM DIF-1 [23], we monitored intracellular cAMP and cGMP levels during this period. DIF-1 at 20 μM increased the intracellular cAMP level by around 20%, and this increase was significant at 4 h (Figure 3A), whereas it did not affect the intracellular cGMP level during incubation for 3 h (Figure 3B). Although the present results (Figure 3A) originated from representative experiments, they are in good agreement with the effect of DIF-1 on intracellular cAMP level in K562 human leukemia cells [26].

### 2.3. Effects of PDE Inhibitors on Glucose Uptake in Confluent 3T3-L1 Cells

**Figure 4:**
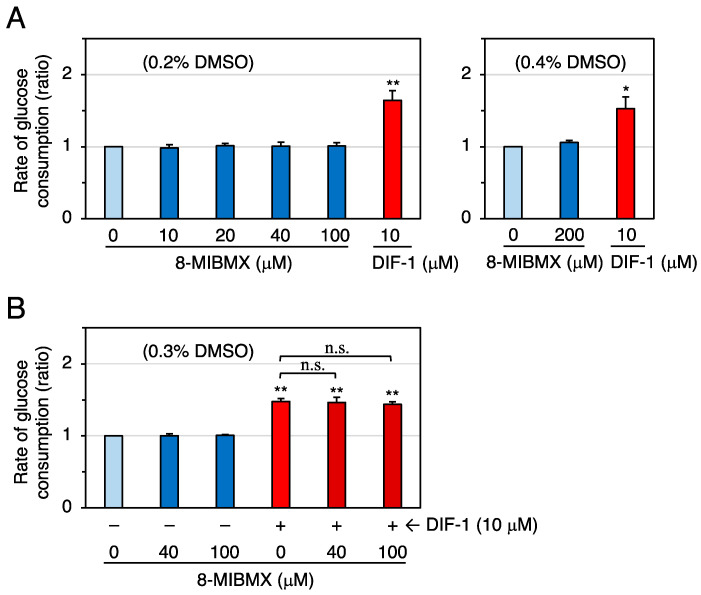
(**A**) Effects of 8-MIBMX and DIF-1 on glucose uptake in confluent 3T3-L1 cells. Cells were incubated for 16–20 h in the presence of 0.2% or 0.4% DMSO (vehicle) and the indicated concentrations of 8-MIBMX or DIF-1, and the rate of glucose consumption was assessed. The data are mean ± SD of three independent experiments. * *p* < 0.05, ** *p* < 0.01 versus control (by *t*-test). (**B**) Combinatorial effects of 8-MIBMX and DIF-1 on glucose uptake. Cells were incubated for 16–20 h in the presence of 0.3% DMSO (vehicle) and the indicated concentrations of 8-MIBMX, DIF-1, or both, and the rate of glucose consumption was assessed. The data are mean ± SD of four independent experiments. ** *p* < 0.01 versus control (by *t*-test); n.s., not significant (by ANOVA).

To further assess whether PDE1 inhibition is involved in DIF-1-promoted glucose uptake, we examined the effects of the specific PDE1 inhibitor 8-methoxymethyl-3-isobutyl-1-methylxanthine (8-MIBMX) on glucose uptake in confluent 3T3-L1 cells (Figure 4). At 10–200 μM, 8-MIBMX did not significantly affect the rate of glucose uptake (Figure 4A), and DIF-1 (10 μM) and 8-MIBMX (40 or 100 μM) showed no additive effects (Figure 4B).

**Figure 5:**
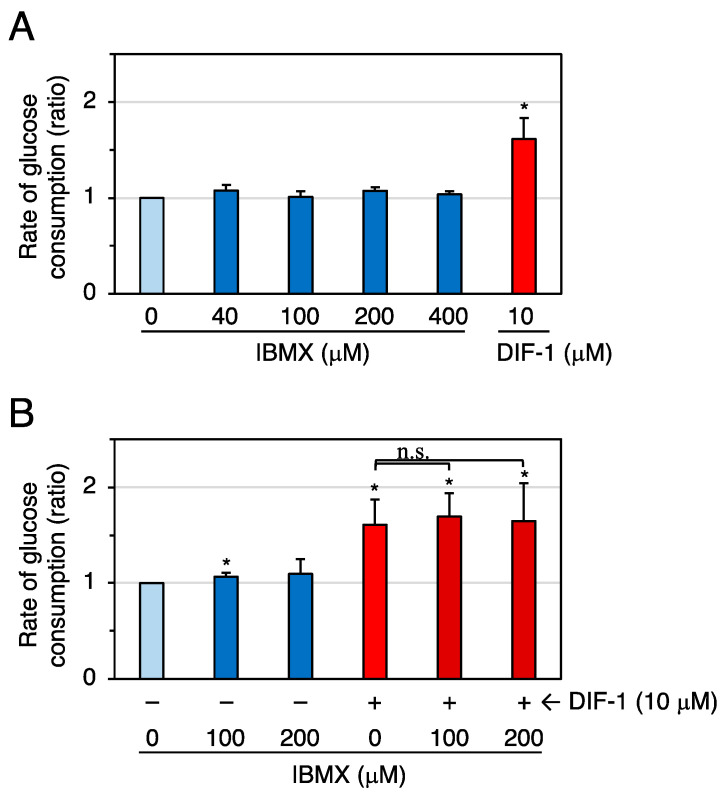
(**A**) Effects of IBMX and DIF-1 on glucose uptake in confluent 3T3-L1 cells. Cells were incubated for 16–20 h in the presence of 0.2% DMSO (vehicle) and the indicated concentrations of IBMX or DIF-1, and the rate of glucose consumption was assessed. The data are mean ± SD of three independent experiments. * *p* < 0.01 versus control (by *t*-test). (**B**) Combinatorial effects of IBMX and DIF-1 on glucose uptake. Cells were incubated for 16–20 h in the presence of 0.2% DMSO (vehicle) and the indicated concentrations of MIBMX, DIF-1, or both, and the rate of glucose consumption was assessed. The data are mean ± SD of four independent experiments. * *p* < 0.01 versus control (by *t*-test); n.s., not significant (by ANOVA).

We next examined the effects of the broad-spectrum PDE inhibitor 3-isobutyl-1-methylxanthine (IBMX) on glucose uptake in confluent 3T3-L1 cells (Figure 5). At 40–400 μM, IBMX did not significantly affect the rate of glucose uptake in the experiment shown in Figure 5A, but, in another experiment (Figure 5B), 100 μM IBMX significantly, albeit slightly, promoted glucose uptake. DIF-1 (10 μM) and IBMX (100–200 μM) showed no additive effects (Figure 5B).

### 2.4. Effects of 8-Bromo-cAMP (Br-cAMP) and 8-Bromo-cGMP (Br-cGMP) on Glucose Uptake in Confluent 3T3-L1 Cells

**Figure 6:**
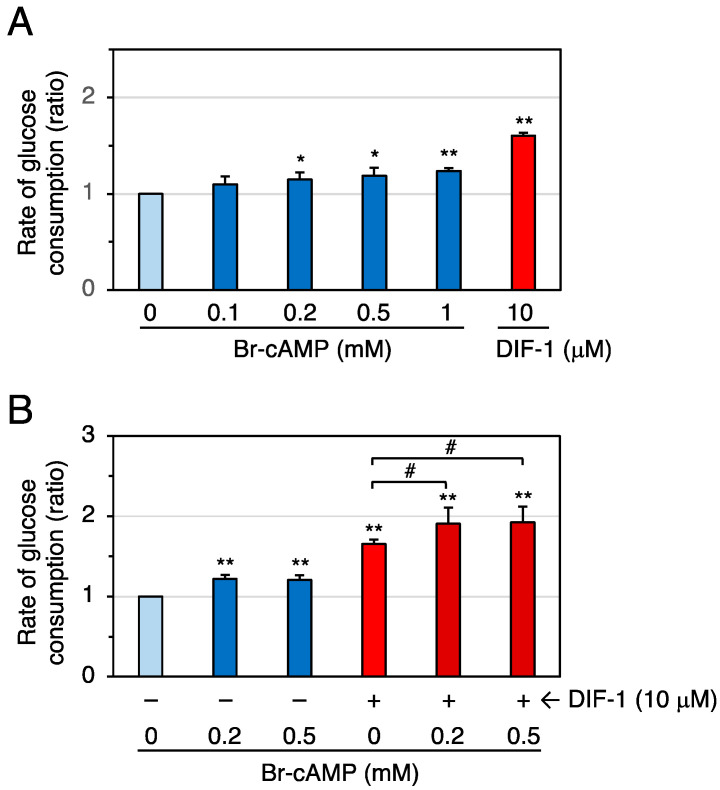
(**A**) Effects of Br-cAMP and DIF-1 on glucose uptake in confluent 3T3-L1 cells. Cells were incubated for 16–20 h in the presence of 0.1% DMSO (vehicle) and the indicated concentrations of Br-cAMP or DIF-1, and the rate of glucose consumption was assessed. The data are mean ± SD of three independent experiments. * *p* < 0.05, ** *p* < 0.01 versus control (by *t*-test). (**B**) Combinatorial effects of Br-cAMP and DIF-1 on glucose uptake. Cells were incubated for 16–20 h in the presence of 0.1% DMSO (vehicle) and the indicated concentrations of Br-cAMP, DIF-1, or both, and the rate of glucose consumption was assessed. The data are mean ± SD of five independent experiments. ** *p* < 0.01 versus control (by *t*-test); ^#^ *p* < 0.05 (by ANOVA).

**Figure 7:**
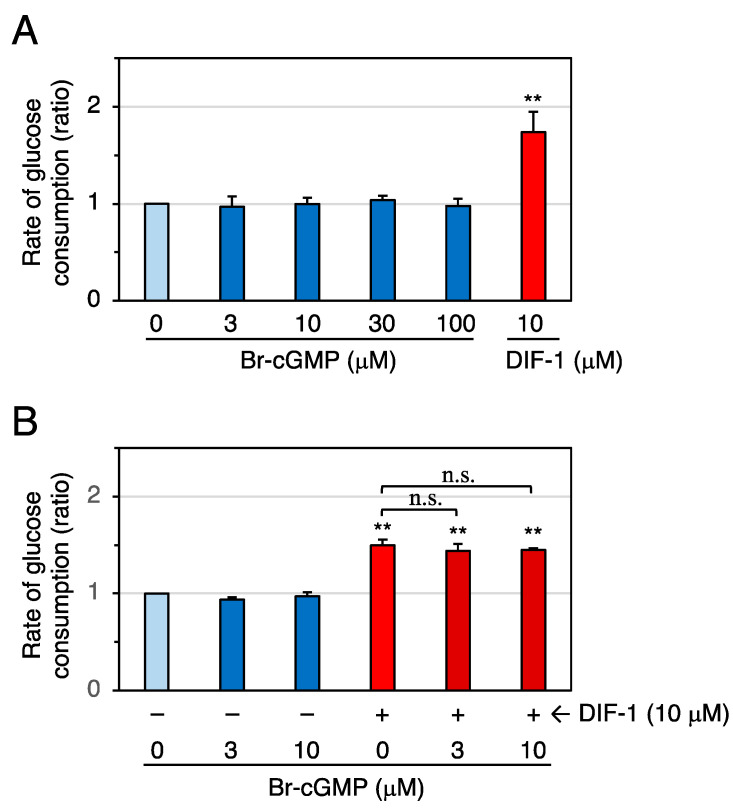
(**A**) Effects of Br-cGMP and DIF-1 on glucose uptake in confluent 3T3-L1 cells. Cells were incubated for 16–20 h in the presence of 0.1% DMSO (vehicle) and the indicated concentrations of Br-cGMP or DIF-1, and the rate of glucose consumption was assessed. The data are mean ± SD of three independent experiments. ** *p* < 0.01 versus control (by *t*-test). (**B**) Combinatorial effects of Br-cGMP and DIF-1 on glucose uptake. Cells were incubated for 16–20 h in the presence of 0.1% DMSO (vehicle) and the indicated concentrations of Br-cGMP, DIF-1, or both, and the rate of glucose consumption was assessed. The data are mean ± SD of three independent experiments. ** *p* < 0.01 versus control (by *t*-test); n.s., not significant (by ANOVA).

We then examined the effects of the cell-membrane-permeable cAMP analog Br-cAMP on glucose uptake in confluent 3T3-L1 cells (Figure 6). At 0.2–1 mM, Br-cAMP significantly promoted glucose uptake (Figure 6A), and DIF-1 (10 μM) and Br-cAMP (0.2–0.5 mM) showed an additive effect (Figure 6B). We next examined the effects of the cell-membrane-permeable cGMP analog Br-cGMP on glucose uptake in confluent 3T3-L1 cells (Figure 7). At 3–100 μM, Br-cGMP did not significantly affect the rate of glucose consumption (Figure 7A). DIF-1 (10 μM) and Br-cGMP (3 or 10 μM) showed no additive effects (Figure 7B). These results suggest that an increase in intracellular cAMP, but not intracellular cGMP, promotes glucose uptake in 3T3-L1 cells.

### 2.5. Effects of Forskolin on Glucose Uptake in Confluent 3T3-L1 Cells

**Figure 8:**
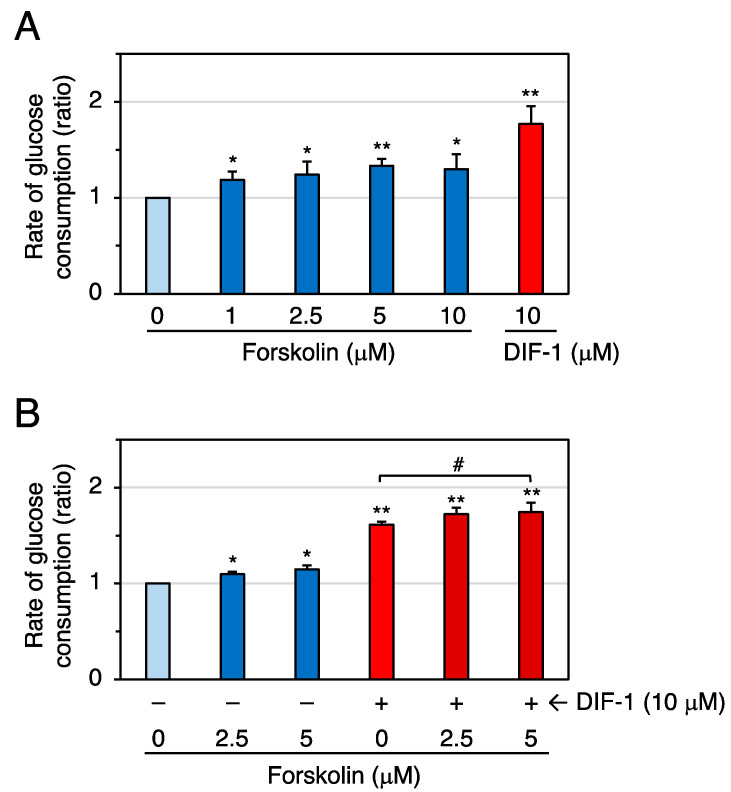
(**A**) Effects of forskolin and DIF-1 on glucose uptake in confluent 3T3-L1 cells. Cells were incubated for 16–20 h in the presence of 0.2% DMSO (vehicle) and the indicated concentrations of forskolin or DIF-1, and the rate of glucose consumption was assessed. The data are mean ± SD of three independent experiments. * *p* < 0.05, ** *p* < 0.01 versus control (by *t*-test). (**B**) Combinatorial effects of forskolin and DIF-1 on glucose uptake. Cells were incubated for 16–20 h in the presence of 0.2% DMSO (vehicle) and the indicated concentrations of forskolin, DIF-1, or both, and the rate of glucose consumption was assessed. The data are mean ± SD of four independent experiments. * *p* < 0.05, ** *p* < 0.01 versus control (by *t*-test); ^#^ *p* < 0.05 (by ANOVA).

To prove that an increase of intracellular cAMP can promote glucose uptake, we examined the effects of the adenylate cyclase activator forskolin on glucose uptake in confluent 3T3-L1 cells (Figure 8). At 1–10 μM, forskolin significantly promoted glucose uptake (Figure 8A), and DIF-1 (10 μM) and forskolin (5 μM) showed an additive effect (Figure 8B). At 20 μM, forskolin appeared to be slightly toxic to the cells as it changed cell morphology and made the boundaries between cells clearer after 20 h incubation. Since forskolin is expected to increase intracellular cAMP levels [33,34], our results suggest that an increase in intracellular cAMP levels can promote glucose uptake in confluent 3T3-L1 cells.

### 2.6. Effects of Mitochondrial Uncoupler on Glucose Uptake in Confluent 3T3-L1 Cells

**Figure 9:**
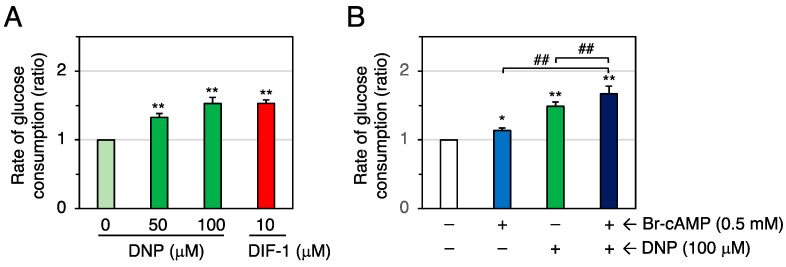
(**A**) Effects of DNP and DIF-1 on glucose uptake in confluent 3T3-L1 cells. Cells were incubated for 16–20 h in the presence of 0.1% DMSO (vehicle) and the indicated concentrations of DNP or DIF-1, and the rate of glucose consumption was assessed. The data are mean ± SD of three independent experiments. ** *p* < 0.01 versus control (by *t*-test). (**B**) Combinatorial effects of DNP and Br-cAMP on glucose uptake. Cells were incubated for 16–20 h in the presence of 0.1% DMSO (vehicle) and the indicated concentrations of DNP and/or Br-cAMP, and the rate of glucose consumption was assessed. The data are mean ± SD of four independent experiments. * *p* < 0.05, ** *p* < 0.01 versus control (by *t*-test); ^##^ *p* < 0.01 (by ANOVA).

To address the possibility that DIF-1 promotes glucose uptake via both mitochondrial uncoupling and intracellular cAMP elevation, we examined the combinatorial effects of the mitochondrial uncoupler dinitrophenol (DNP) and Br-cAMP on glucose uptake in confluent 3T3-L1 cells (Figure 9). DNP (50 or 100 μM) increased the rate of glucose consumption (Figure 9A), as described previously [24]. Br-cAMP (0.5 mM) and DNP (100 μM) showed an additive effect (Figure 9B). These results suggest that DIF-1 may promote glucose uptake via both mitochondria-dependent and cAMP-dependent pathways in 3T3-L1 cells.

### 2.7. Effects of PDE Inhibitors and Cellular cAMP and cGMP Levels on Cell Growth of 3T3-L1 and HeLa Cells

**Figure 10:**
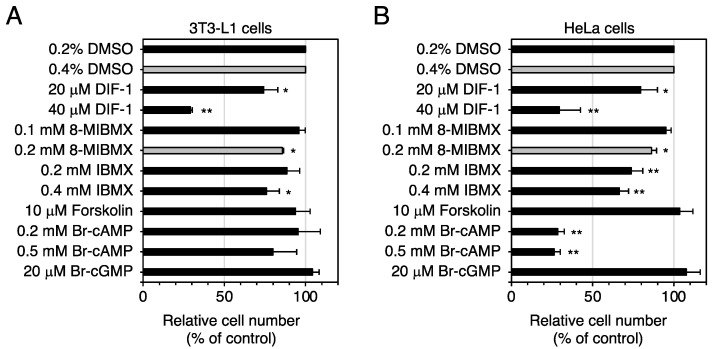
Effects of the compounds affecting cAMP/cGMP levels on the growth of 3T3-L1 (**A**) and HeLa cells (**B**). Growing cells were incubated for 3 days in the presence of the indicated compounds, and relative cell number was determined. All media contained 0.2% (black bars) or 0.4% (gray bars) DMSO (vehicle). The data are mean ± SD of three independent experiments. * *p* < 0.05; ** *p* < 0.01 versus control (by *t*-test).

We compared the effects of 8-MIBMX, IBMX, forskolin, Br-cAMP, and Br-cGMP on cell growth in 3T3-L1 cells, using human cervical cancer HeLa cells as a reference (Figure 10). In 3T3-L1 cells, forskolin at 10 μM, Br-cAMP at 0.2 or 0.5 mM, and Br-cGMP at 20 μM had no significant effect, whereas DIF-1 at 20 or 40 μM, 8-MIBMX at 0.2 mM, and IBMX at 0.4 mM significantly suppressed cell growth (Figure 10A). These results suggest that the effect of increases in intracellular cAMP or cGMP on 3T3-L1 cell growth is small, if there is any at all.

In HeLa cells, forskolin at 10 μM had no effect on cell growth, whereas Br-cAMP at 0.2 or 0.5 mM greatly suppressed it (Figure 10B). These results agree well with previous observations that Br-cAMP and another membrane-permeable cAMP analog, dibutyryl cAMP, suppress growth of HeLa cells and some other tumor cells [35,36,37,38]. The reason for the lack of effect of forskolin is presently unknown. As intracellular cAMP levels are determined by the balance of the activities of adenylate cyclases and PDEs, this forskolin concentration might be insufficient to suppress the growth of HeLa cells but sufficient to suppress (by 25–50%) growth of some other mammalian cells such as mouse neuroblastoma/rat glioma hybrid cells, NG108-15 [39], or non-small-cell lung cancer cells, H1299 and A549 [40].

## 3. Discussion

### 3.1. DIF-1 as a Lead Compound for Anticancer and Anti-Diabetic Drug Development

DIF-1 (Figure 1A) was originally isolated as a stalk cell differentiation-inducing factor in *D. discoideum* [4]. Later, it was shown that DIF-1 and its derivatives possess antitumor (anti-proliferative and anti-metastatic) activities in mammalian cells in vitro and in vivo [9,10,11,12,13,14,15,16,17,18,19,20,21,22]. Studies conducted since 2007 have shown that DIF-1 also has strong glucose-uptake-promoting activities in mammalian cells in vitro and possibly in vivo and is thus a promising lead for the development of anti-obesity and anti-diabetes drugs [20,23,24,25,32].

In 3T3-L1 cells, DIF-1 had a strong glucose-uptake-promoting activity (particularly at 20 μM) and a low anti-proliferative activity (Figure 2). These data agree well with DIF-1 being a good lead for the development of anti-obesity and anti-diabetic drugs.

### 3.2. Mechanism of Action of DIF-1: Involvement of PDE1

AMPK is a heterotrimeric protein composed of α, β, and γ subunits; when the cellular AMP-to-ATP ratio is increased by metabolic stress, the α subunit is phosphorylated, leading to AMPK activation [41,42,43,44]. We have revealed so far that DIF-1 functions as a mitochondrial uncoupler and can promote glucose uptake, at least in part via a mitochondria- and AMPK-dependent pathway, in 3T3-L1 cells [24] (Figure 1B). Some unknown pathway(s) may also mediate DIF-1 function (Figure 1B) because neither the AMPK inhibitor compound C nor RNAi for AMPK completely blocks the DIF-1-induced glucose uptake in 3T3-L1 cells, and the known uncoupler DNP is not as effective as DIF-1 at similar concentrations [24].

Here, we focused on DIF-1 as a direct inhibitor of PDE1 [26], which can degrade cAMP or cGMP; we assumed that DIF-1 may function, at least in part, via PDE1 inhibition and subsequent increases in intracellular cAMP and/or cGMP levels (Figure 1B). In this study, we showed that the intracellular cAMP content was increased by about 20% on average in confluent 3T3-L1 cells cultured with 20 μM DIF-1 for 4 h (Figure 3A), whereas no significant change in cGMP content was detected during 3 h incubation with 20 μM DIF-1 (Figure 3B). Although our results were obtained from representative experiments, the results agree well with a previous observation that 20 μM DIF-1 increases intracellular cAMP content by about 20% in K562 human leukemia cells [26]. Of interest here is that progesterone is thought to induce germinal vesicle breakdown (meiosis) by inhibiting membrane-bound adenylate cyclase [45,46] and thereby decreasing intracellular cAMP concentration by about 20% in *Xenopus* oocytes [47], and the progesterone-induced cAMP decrease and germinal vesicle breakdown can be blocked with 30 μM DIF-1 [48]. Therefore, the DIF-1-induced slight increase in intracellular cAMP observed in the current study may have functional effects.

Contrary to our expectation, the specific PDE1 inhibitor 8-MIBMX at up to 0.2 mM did not markedly affect glucose uptake in confluent 3T3-L1 cells (Figure 4), and the glucose-uptake-promoting effect of the broad-range PDE inhibitor IBMX at up to 0.4 mM (Figure 5) was very weak or absent. Although the membrane permeability of 8-MIBMX and IBMX in confluent 3T3-L1 cells is not known, both inhibitors are effective at 0.1–0.2 mM in other in vitro cell culture systems [49,50]. Thus, our results suggest that PDEs, including PDE1, may not be involved in DIF-1-induced glucose uptake. On the other hand, since 8-MIBMX in the same concentration range significantly suppressed growth of 3T3-L1, HeLa (Figure 10), and human K562 leukemia cells [26], DIF-1 may suppress cell growth, at least in part, by inhibiting PDE1 in mammalian cells. Note that some inhibitors for PDEs, including PDE1, are expected to have therapeutic potential in cancer treatment [51,52,53,54,55].

### 3.3. Involvement of Intracellular cAMP in the Actions of DIF-1

**Figure 11:**
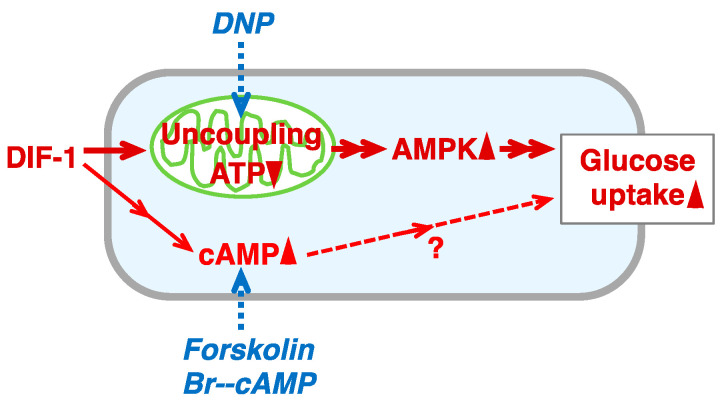
Proposed scheme for the action of DIF-1 in 3T3-L1 cells. DIF-1 promotes glucose uptake, in part via a mitochondria- and AMPK-dependent pathway [23,24] and possibly also via an increase in intracellular cAMP level (this study). The mitochondrial uncoupler DNP can stimulate the mitochondria- and AMPK-dependent pathway and thereby promote GLUT1 translocation to the plasma membrane (PM) and glucose uptake [24], whereas Br-cAMP (membrane-permeable analog of cAMP) and forskolin (adenylate cyclase activator) increase the intracellular cAMP level and thus mimic the effect of DIF-1 (this study). Note that an increase in intracellular cAMP has been shown to induce GLUT1 translocation to the plasma membrane and promote glucose uptake in 3T3-L1 adipocytes and brown fat cells [29,30].

The membrane-permeable cAMP analog Br-cAMP at 0.2–1 mM significantly promoted glucose uptake by 15–25% (Figure 6A), and 0.2–0.5 mM Br-cAMP and 10 μM DIF-1 showed additive effects (Figure 6B). These results agree with previous observations that 0.5–1 mM Br-cAMP promotes glucose uptake in 3T3-L1 adipocytes [29] and mouse brown adipose tissue [30], suggesting that intracellular cAMP is involved in DIF-1-induced glucose uptake (Figure 11). Although the membrane-permeable cGMP analog Br-cGMP is functional at 1–100 μM because it induces relaxation of isolated rat thoracic arteries [56], 3–100 μM Br-cGMP did not promote glucose uptake (Figure 7A), and 3–10 μM Br-cGMP and 10 μM DIF-1 showed no additive effects in confluent 3T3-L1 cells (Figure 7B), suggesting that intracellular cGMP may not be involved in DIF-1-induced glucose uptake.

The adenylate cyclase activator forskolin at 1–10 μM promoted glucose uptake, and 5 μM forskolin and 10 μM DIF-1 showed additive effects in confluent 3T3-L1 cells (Figure 8). The mitochondrial uncoupler DNP at 50 or 100 μM promoted glucose uptake, and DNP (100 μM) and Br-cAMP (0.5 mM) showed an additive effect in confluent 3T3-L1 cells (Figure 9). Overall, our results suggest that DIF-1 may promote glucose uptake, at least in part, via an increase in intracellular cAMP levels (Figure 11).

We intend to further elucidate how DIF-1 increases the intracellular cAMP level and how elevated cAMP promotes glucose uptake in 3T3-L1 cells, which we expect will contribute to the development of novel anti-obesity and anti-diabetes agents.

## 4. Materials and Methods

### 4.1. Cells and Reagents

Mouse 3T3-L1 fibroblasts and human cervical cancer HeLa cells were used. Cells were maintained at 37 °C (5% CO_2_) in DMEM-HG (Dulbecco’s Modified Eagle’s Medium containing a high concentration (4.5 mg/mL) of glucose (Fujifilm Wako Pure Chemical Corporation, Osaka, Japan) supplemented with 75 μg/mL penicillin, 50 μg/mL streptomycin, and 10% (*v*/*v*) heat-inactivated fetal bovine serum (FBS)). DIF-1 was synthesized as previously described [15], dissolved in DMSO at 5–20 mM, and stored at −20 °C. IBMX, 8-MIBMX, Br-cAMP, and Br-cGMP were obtained from Sigma (St. Louis, MO, USA). Forskolin and DNP were from Fujifilm Wako Pure Chemical Corporation. DMSO solutions of IBMX (20–200 mM), 8-MIBMX (5–50 mM), forskolin (1–10 mM), and DNP (50–100 mM) and aqueous solutions of Br-cAMP (100 mM) and Br-cGMP (1–10 mM) were prepared and stored at −20 °C.

### 4.2. Assessment of Glucose Consumption (Uptake) in 3T3-L1 Cells

The rate of glucose consumption was assessed primarily as described by Omata et al. [23]. 3T3-L1 cells were incubated in DMEM-HG (1 mL per well) for 3–4 days in a 12-well plate (Corning, New York, NY, USA) until the cells reached confluency. The cells were then incubated for 16–20 h with the additives in 1 mL of fresh DMEM-MG (DMEM containing a medium concentration (2 mg/mL) of glucose supplemented with the antibiotics, 10% FBS, and 10 mM HEPES-NaOH (pH 7.4)). The glucose concentration in DMEM-MG aliquots was measured with a hand-held glucose monitor, GlucCell^TM^, and its sensor chips, Glucose Test Strips (Cesco Bioengineering Co., Ltd., Taichung, Taiwan), and was used to calculate the rate of glucose consumption.

### 4.3. Cell Growth Assay

Cells (3T3-L1 or HeLa) were incubated in 12-well plates in DMEM-HG (5 × 10^3^ cells in 1 mL per well) overnight until they adhered to the bottom. The medium was replaced with 1 mL of fresh DMEM-HG containing additives, and the cells were grown for 3 days. The medium was removed, and the cells were washed with 0.5 mL of 10 mM phosphate-buffered saline (pH 7.4) and incubated in 0.5 mL of DMEM-HG without phenol red (Fujifilm Wako Pure Chemical Corporation) containing 5% Alamar blue (a cell number indicator; Fujifilm Wako Pure Chemical Corporation) until the color of the medium changed from blue to reddish purple. Cell number relative to control was determined by measuring absorbance at 570 nm (reference at 595 nm).

### 4.4. Measurement of Intracellular cAMP Levels

Confluent 3T3-L1 cells were incubated with 1 mL of DMEM-MG containing 20 μM DIF-1 in 12-well plates. At the beginning of the incubation, cells were collected from one well, and cell number was determined by counting the cells. At the time points indicated in the figures, 0.8 mL of each incubation medium was removed (0.2 mL remained) from the remaining wells, and 0.2 mL of 3.5% HClO_4_ solution was added to each well. Each cell suspension (0.4 mL) was collected into a 1.5 mL microcentrifuge tube and stored at −20 °C until cAMP assay. The lysate was neutralized by adding 100 μL of 50% saturated KHCO_3_, and the cAMP concentration was determined with a Cyclic AMP Select ELISA Kit (Cayman Chemical, Ann Arbor, MI, USA) according to the manufacturer’s instructions.

### 4.5. Measurement of Intracellular cGMP Levels

Confluent 3T3-L1 cells were incubated with 5 mL of DMEM-MG containing 20 μM DIF-1 in 3.5 cm dishes. At the beginning of the incubation, cells were collected from one dish, and cell number was determined by counting the cells. At the time points indicated in the figures, the media were removed from the remaining dishes, and each dish was washed with 2 mL of 10 mM phosphate-buffered saline, after which 0.2 mL of 1.75% HClO_4_ solution was added to each dish. A 0.2 mL aliquot of each cell suspension was collected into a 1.5 mL microcentrifuge tube and stored at −20 °C until cGMP assay. The lysate was neutralized by adding 100 μL of 50% saturated KHCO_3_, and the cGMP concentration was determined with a Cyclic GMP Select ELISA Kit (Cayman Chemical) according to the manufacturer’s instructions.

### 4.6. Statistical Analyses

Statistical analyses were performed by Student’s *t*-test (two tailed, unpaired) or one-way analysis of variance (ANOVA) followed by Tukey’s multiple-comparison test. Values were considered to be significantly different when the *p* value was less than 0.05.

## 5. Conclusions

To elucidate the mechanism(s) underlying the glucose-uptake-promoting effect of DIF-1, we analyzed the relationship between intracellular cAMP and cGMP levels and DIF-1 activity in confluent mouse 3T3-L1 fibroblasts. It was shown here that DIF-1 increased the level of intracellular cAMP but did not affect the level of intracellular cGMP, whereas an increase in intracellular cAMP mimicked by Br-cAMP or induced by forskolin promoted glucose uptake. Our results suggest that DIF-1 may play a role in promoting glucose uptake via an increase in intracellular cAMP level in these cells.

## Data Availability

Some or all data generated or analyzed during this study are included in this published article.
